# Collaborative pharmacist prescribing in community heart failure clinics: a pre-post intervention comparison

**DOI:** 10.1007/s11096-026-02186-0

**Published:** 2026-07-17

**Authors:** Matthew Percival, Laetitia Hattingh, Rohan Jayasinghe, James Millhouse, Joanne Crook, Carl De Wet, Ian Hughes, Christopher Freeman

**Affiliations:** 1 School of Pharmacy and Medical Sciences, Griffith University, Southport, QLD Australia; 2 School of Pharmacy, The University of Queensland, Brisbane, QLD Australia; 3 Integrated Care Services, Gold Coast Hospital and Health Service (GCHHS), Southport, QLD Australia; 4 Allied Health Research, GCHHS, Southport, QLD Australia; 5 Department of Pharmacy, GCHHS, Southport, QLD Australia; 6 Department of Cardiology, GCHHS, Southport, QLD Australia; 7 Southwest Hospital and Health Service, Roma, QLD Australia; 8 Office for Research Governance and Development, Gold Coast University Hospital, Southport, Australia

**Keywords:** Collaborative pharmacist medication prescribing, Drug therapy, Heart failure, Medication therapy management, Pharmaceutical services

## Abstract

**Introduction:**

Guideline-directed medical therapy (GDMT) improves outcomes in heart failure, yet many patients remain undertreated or are not titrated to optimal doses. International evidence shows pharmacist-led heart failure clinics improve GDMT prescribing and reduce hospitalisations, but data in Australian settings are limited.

**Aim:**

To evaluate whether incorporating a collaborative pharmacist medication prescribing model within a heart failure service improved optimisation of guideline-directed medical therapy (GDMT) in patients with heart failure and ejection fraction < 50% compared with pre-implementation usual care, and to evaluate impacts on service efficiency, hospitalisation, mortality, medication beliefs, and health-related quality of life.

**Method:**

An interrupted time series analysis compared 12-month pre- and post-implementation periods following establishment of a collaborative pharmacist medication prescribing clinic across two community heart failure services. Primary outcomes were the proportion of patients optimised on GDMT at 90 days and at clinic discharge. Secondary outcomes included time between visits, monthly attendance, unscheduled hospitalisations, medication adherence and health related quality of life (HRQoL) assessed with the EuroQoL five-dimension five-level health status instrument visual analogue scale (EQ-VAS) and the Kansas City Cardiomyopathy Questionnaire (KCCQ-12).

**Results:**

A total of 302 patients with ejection fraction < 50% were included, with 98 and 204 patients in the pre- and post-implementation cohort respectively. Overall, 285 patients (94.4%) had a left ventricular ejection fraction ≤ 40%, with similar proportions between cohorts (94.6% vs. 93.9%; *p* = 1.000). Optimisation of GDMT increased from 19.3% (n = 19) to 40.2% (n = 82) at 90 days (adjusted OR = 2.6, *p* = 0.001), and from 43.9% (n = 43) to 88.2% (n = 180) at discharge (adjusted OR = 13.2, *p* < 0.001). Median time between visits decreased from 49 to 28 days (*p* < 0.001), while mean monthly attendance increased from 9 to 16 (*p* < 0.001). HRQoL scores increased within the post-implementation group, measured by the KCCQ-12 (74.0 → 84.8, *p* < 0.001) and EQ-VAS (73.0 → 79.5, *p* = 0.004). Medication adherence remained high according to self-reported Medication Adherence Report Scale (MARS) scores. No change in hospitalisations or mortality was observed.

**Conclusion:**

Introducing a collaborative pharmacist medication prescribing clinic to usual care was associated with higher rates of GDMT optimisation and increased clinic throughput. These findings support the feasibility of collaborative prescribing models to facilitate GDMT optimisation within Australian health services.

## Impact statements

- Collaborative pharmacist prescribing may improve access to guideline-directed medical therapy optimisation for patients with heart failure.
- Embedding pharmacist prescribing within multidisciplinary heart failure services may support more timely medication titration and improve clinic throughput.
- This model may help address workforce and prescriber access limitations, particularly in rural and remote settings.

## Introduction

Patient prognosis from heart failure is worse than most cancers, with 12% 30-day and 31% 1-year post admission mortality, and annual costs to the Australian economy of almost $2.7 billion [1]. Guideline directed medical therapy (GDMT), referred to as the ‘four pillars’ of heart failure therapy, includes: (i) renin-angiotensin system inhibitors, (ii) select beta-blockers, (iii) mineralocorticoid receptor antagonists, (iv) sodium glucose co-transporter 2 inhibitors [2–4], with demonstrated reductions in morbidity and mortality when prescribed in patients with heart failure with reduced ejection fraction (EF) < 50%. However, in real world settings, therapeutic inertia, patient medication adherence/tolerance issues and medical workforce challenges frequently prevent initiation or uptitration to effective doses as demonstrated in prior evidence on pharmacist prescribing. [5–7]

International studies demonstrate that pharmacist-led heart failure clinics improve prescribing of GDMT [8–15] with reductions in hospitalisations (Absolute Risk Reduction [ARR] 20%, *p* = 0.008) [11, 16], mortality (ARR 11%, *p *= 0.010) [12], and health care savings (estimated > USD 50,000 annual revenue generated from 0.2 full time equivalent pharmacist) [10]. In pharmacist-led heart failure clinics, pharmacists work either independently (as independent prescribers) or collaboratively (collaborative pharmacist medication prescribing) with doctors, to optimise doses of GDMT to manage heart failure, provide patient follow up, as well as patient education and counselling. [10] In Australia, legislation now permits collaborative pharmacist medication prescribing in hospitals. Under this model, an authorised pharmacist and a medical or nurse practitioner jointly review the patient’s condition and jointly agree on a management plan. [17]

The Gold Coast Hospital and Health Service (GCHHS), Southeast Queensland, Australia, provides acute, sub-acute and community care across more than 20 facilities for a population of over 711,000 people in the Gold Coast and northern New South Wales. It provides a cardiology advanced trainee-led heart failure clinic in two community centre-based clinics, targeted at optimising GDMT with multidisciplinary support including pharmacists, nursing staff and allied health. In Australia, advanced trainees operate as registrars, managing patients with increasing independence while practising under the broader governance and supervision of consultant cardiologists. Within the GCHHS heart failure service, advanced trainees have operated as the primary attending clinicians since the service commenced in 2006 and therefore held responsibility for day-to-day patient management within the community heart failure clinic. Although patients were managed independently by the advanced trainee in the heart failure clinic, senior cardiology specialists remained available for consultation and escalation where additional clinical guidance or review was required. The clinic receives up to 500 referrals per year, with each patient often requiring multiple visits. However, the clinic only has capacity to provide services to up to 12 patients each week limited by staffing restrictions. To meet this gap in demand, a collaborative pharmacist medication prescribing model of care was developed to optimise GDMT in patients with heart failure.

### Aim

*Primary aim*: To determine whether introduction of a collaborative pharmacist medication prescribing model increased the proportion of patients with heart failure and ejection fraction < 50% optimised on GDMT at 90 days and at discharge compared with a 12-month pre-implementation period.

*Secondary aims*: to compare (i) service efficiency (patients seen per month; time between visits); (ii) 30-day and 6-month HF-related and all-cause hospitalisations and mortality; and, in post-implementation patients, (iii) evaluate baseline to discharge changes in medication beliefs and health-related quality of life (HRQoL) in the post-implementation cohort.

## Method

### Study design and setting

We conducted a quasi-experimental before–after study with interrupted time-series (ITS) analysis across two community heart failure clinics at the Helensvale community health centre (established 2006) and Robina Health Precinct (2011), within the Gold Coast Hospital and Health Service.

The collaborative pharmacist medication prescribing model was implemented as routine care in March 2023 to support the advanced trainee led heart failure clinic. Accordingly, the post-implementation cohort included all patients referred to the heart failure service during the study period, rather than only patients selectively referred to the collaborative pharmacist medication prescribing model. The results and outcomes of the study reflect the combined efforts of both the pharmacist and doctor model of care. The pre-implementation period chosen for analysis was 1 December 2021 to 31 December 2022 (52 weeks); post-implementation: 1 March 2023 to 31 March 2024 (54 weeks). These time periods were separated by a 2-month washout (Jan – Feb 2023) to avoid patient overlap. Outcomes were compared between periods on an intention-to-treat basis.

### Patient criteria, recruitment and data collection

- Inclusion:
  - Adults ≥ 18 years with heart failure and left ventricular ejection fraction (LVEF) < 50%, encompassing both heart failure with reduced ejection fraction (HFrEF; LVEF ≤ 40%) and heart failure with mildly reduced ejection fraction (HFmrEF; LVEF 41–49%),
  - who underwent active guideline-directed medical therapy (GDMT) optimisation through the HF titration clinic.
- Exclusion:
  - Patients unable to attend follow-up appointments,
  - patients discharged following initial review without entering the HF titration clinic optimisation pathway,
  - and patients transferred back to general practitioner or external outpatient management prior to completion of planned optimisation

Patients already established on optimal or maximally tolerated GDMT at initial clinic review were excluded from optimisation analyses, as the study aimed to evaluate medication optimisation achieved through the HF titration clinic intervention rather than pre-existing therapy optimisation. Routine clinical data (demographics, vitals (blood pressure, heart rate, weight), labs (renal function, potassium) and GDMT doses were collected under a waiver of consent in pre-implementation and post-implementation arms. Informed, written consent was obtained for non-routine questionnaires in the post-implementation arm: Medication Adherence Report Scale (MARS), Beliefs about Medicines Questionnaire (BMQ), Kansas City Cardiomyopathy Questionnaire-12 (KCCQ-12), and EQ-5D-5L. These self-reported tools describe medication adherence behaviours, beliefs, and HRQoL (baseline-to-discharge changes; post-implementation only due to intervention timing), acknowledging limitations of social desirability/recall bias and ceiling effects. [18–26] For our research, these tools were used as a means of describing attitudes or behaviours rather than attempting to precisely quantify adherence or HRQoL.

#### Usual care—i.e., pre-implementation

Patients received 30-min consultations with a cardiology advanced trainee, supported by the multidisciplinary team (clinical nurse/consultant, pharmacist, allied health) until GDMT optimisation, per cardiology advanced trainee discretion.

#### Intervention—i.e., post-implementation

**Fig. 1:**
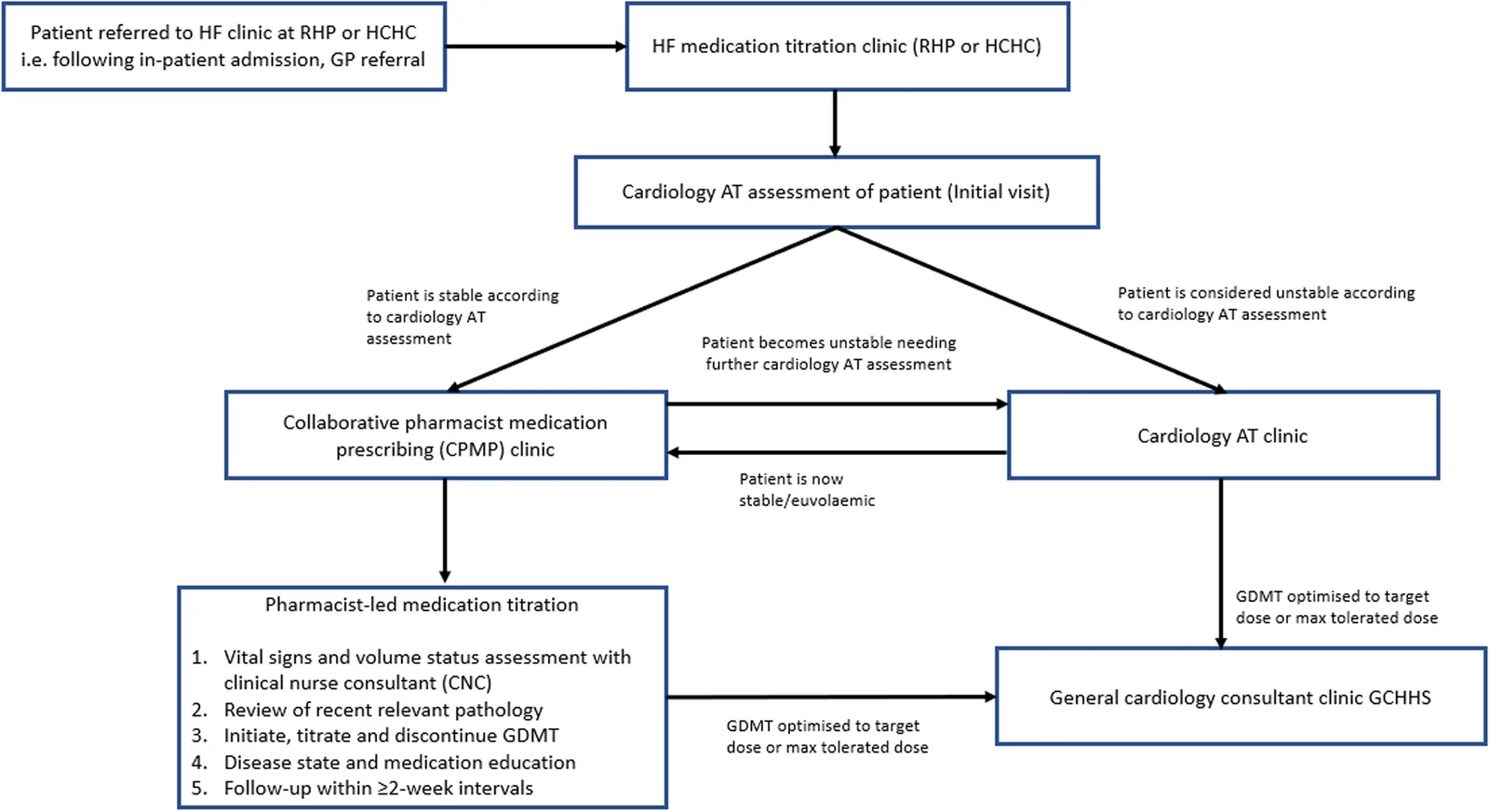
Patient flow through HF clinic post-implementation, n = 204

**Fig. 2:**
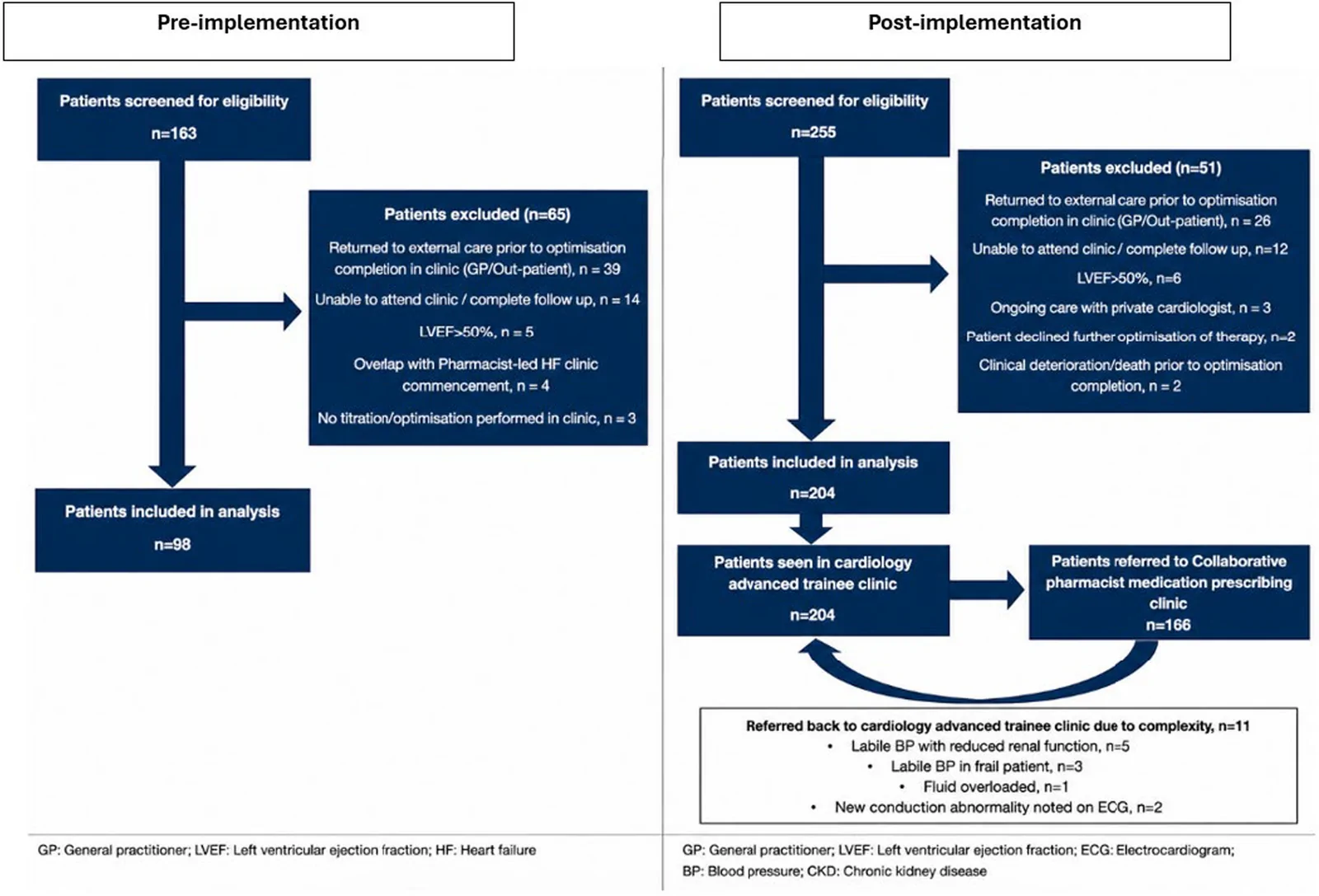
Cohort derivation for pre- and post- implementation

The cardiology advanced trainee continued with the initial 30-min patient review, as they would pre-implementation, but triaged clinically stable patients (euvolaemic/NYHA Class I – III) [27] to the collaborative pharmacist medication prescribing clinic for ongoing GDMT titration. Unstable patients (NYHA Class IV/fluid overloaded) remained under cardiologist care but could transition into the collaborative pharmacist medication prescribing clinic once stabilised. All patients continued to be managed in the heart failure service, thereby maintaining a consistent patient population across the pre- and post-implementation periods. Patients that later became unstable or complex (labile blood pressure, deteriorating renal function, frailty) whilst attending the collaborative pharmacist medication prescribing were escalated back to the cardiology advanced trainee-led clinic following the pharmacist identifying these patients and discussing with the cardiology advanced trainee. Once GDMT was optimised to target or maximally tolerated doses, patients transitioned to a general cardiology consultant follow-up within the same health service. Triage and patient flow are described in Fig. 1, with the cohort derivation for pre- and post- implementation groups presented in Fig. 2.

### Collaborative pharmacist medication prescribing clinic procedures

Clinic referrals began with a 30-min, face-to-face joint intake (pharmacist and clinical nurse consultant), including vitals/pathology review, medication reconciliation, disease/self-care education, and provisional GDMT titration plan aligned to the Advanced Pharmacy Australian Clinical Pharmacy Standards [28]. Plans were approved by the cardiology advanced trainee (who issued prescriptions) with communication updates shared with patients, general practitioners (GP) and community pharmacists (including dose administration aids, DAAs). Escalation occurred to the cardiology advanced trainee for unstable patients/adverse events noted during clinic, with patients optimised on GDMT transitioning back to cardiology consultant follow up.

### Collaborative pharmacist medication prescribing intervention fidelity and roles

A credentialed consultant-level pharmacist (17 years HF experience, credentialed at consultant level in cardiology by the Australian New Zealand College of Advanced Pharmacy (ANZCAP)), delivered consistent care as per TIDieR checklist [29], endorsed by the GCHHS Medicines Advisory Committee. Decisions to commence or uptitrate medications were guided based on previously published parameters and local clinical consensus with the Director of Cardiology and cardiology advanced trainees, detailing acceptable blood pressures or heart rates on which further medication commencement or uptitration could proceed. (Table 1). [8] The same protocol, eligibility criteria, titration thresholds and governance arrangements were applied in all collaborative pharmacist medication prescribing clinic sessions across the health service, with no site-specific variation in pharmacist scope or cardiology oversight.

**Table 1:** Maximal doses of guideline directed medical therapy (GDMT) to manage heart failure with reduced ejection fraction and criteria for determining maximal tolerated doses

Medication		Target/maximal daily dose (mg)	Criteria for determining if at maximal tolerated dose
Angiotensin converting enzyme inhibitors (ACE-I)	Captopril	150	Heart rate (HR) < 65bpm (when assessing beta-blocker) Systolic blood pressure (SBP) ≤ 95mmHg (applies to all 4 medication pillars), SBP > = 100mmHg, but with signs and symptoms of hypotension documented (applies to all 4 medication pillars) Serum potassium (K^+^) > 5mmol/L (applies to ARNIs/ACE-Is/ARBs/MRAs) Adverse drug reaction experienced by patient limiting further uptitration e.g. fatigue, dizziness, genitourinary infection with SGLT2-Is Serum creatinine (Cr) increase > 30% from baseline (applies to ARNIs/ACE-Is/ARBs/MRAs) Clinical judgement of cardiology advanced trainee advises not for further titration as documented in electronic medical record, or verbal discussion
	Enalapril	20	
	Fosinopril	40	
	Lisinopril	30	
	Perindopril (arginine/erbumine)	10/8	
	Quinapril	20	
	Ramipril	10	
	Trandolapril	4	
Angiotensin receptor blockers (ARB)	Candesartan	32	
	Irbesartan	300	
	Olmesartan	40	
	Telmisartan	80	
	Valsartan	320	
Angiotensin receptor Neprilisyn inhibitor (ARNI)	Valsartan/sacubitril	194/206 (I.e., 97/103mg twice daily)	
Beta-blockers	Bisoprolol	10	
	Carvedilol	50 (if weight < 85 kg) 100 (if weight ≥ 85 kg)	
	Metoprolol XR	190	
	Nebivolol	10	
Mineralocorticoid receptor antagonists (MRA)	Spironolactone	25	
	Eplerenone	25	
Sodium glucose co-transporter 2 inhibitors (SGLT2-I)	Dapagliflozin	10	
	Empagliflozin	10	
Example scenario 1: 56 year old male taking Entresto 24/26mg tablets twice daily, bisoprolol 5 mg tablet daily, dapagliflozin 10 mg tablet daily and spironolactone 12.5mg tablet daily. Patient reports no symptoms of dizziness, renal function stable and potassium < 5mmol/L. Blood pressure = 123/86 with no postural drop and heart rate = 60 bpm. Plan = implement proposed plan to increase Entresto to 49/51mg tablet twice a day given stable observations			
Example scenario 2: 86 year old female taking ramipril 5 mg tablet daily, bisoprolol tablet 2.5mg daily, spironolactone 12.5mg tablet daily. No SGLT2-I due to previous genitourinary infection. Patient reports occasional dizziness with postural changes when rushing but ok when slowing down. BP = 100/56mmHg, HR = 55bpm. Plan: discharge as considered on optimal GDMT. Nil further titration of ramipril/spironolactone or bisoprolol due to low blood pressure/symptoms, and HR at target of < 65bpm. Unable to tolerate SGLT2-I			

### Follow-up and optimisation criteria.

In-person follow-ups were scheduled ≥ 2 weeks until target/maximally tolerated GDMT doses were achieved, using the titration parameters and thresholds outlined in Table 1. [11–15]. Patients with documented contraindications or intolerance to certain GDMT classes were assessed based on the therapies they could still receive, so optimisation status reflected appropriate treatment rather than an inability to tolerate specific medications. Patients were classified as achieving optimised GDMT if all clinically indicated and tolerated GDMT classes had been initiated and titrated to either guideline-recommended target doses or maximally tolerated doses according to the predefined criteria outlined in Table 1. We recognise that a systolic blood pressure (SBP) ≤ 95mmHg or heart rate (HR) < 65 beats per minute (bpm) HR should not automatically rule out further GDMT titration in practice. However, because the pre-implementation cohort was reviewed retrospectively, we needed a consistent threshold to assess, based on the available documentation, whether further titration might have been clinically feasible. For this reason, the SBP and HR measures were used pragmatically within the predefined optimisation framework, rather than as a strict treatment rule. This pragmatic approach aligns with previous heart failure optimisation literature using predefined clinical and laboratory thresholds to objectively define maximally tolerated doses when evaluating GDMT. [13]

Patients unable to achieve target doses because of documented contraindications, intolerance, renal dysfunction, hypotension, bradycardia, adverse effects, patient preference, or clinician-directed cessation of titration were still considered optimised if no further evidence-based up-titration was deemed clinically appropriate. Although patients with HFmrEF were included, the cohort predominantly comprised patients with LVEF ≤ 40%, for whom optimisation of the four foundational GDMT therapy classes is guideline-directed standard care. However, patients with HFmrEF were assessed using the same optimisation framework given they represented a small proportion of the overall cohort. Whilst evidence supporting quadruple GDMT is strongest in HFrEF, contemporary guidelines for HFmrEF still consider several foundational therapies reasonable, particularly among patients with LVEF closer to 40%, with strong evidence emerging for SGLT2-I across the heart failure ejection fraction spectrum. [3, 4, 6] Phone follow-ups occurred between visits to assess tolerability.

Outcomes

Primry outcomes

Proportion on optimised (target/max tolerated doses) GDMT at a) 90-days, and b) discharge.

Secondary outcomes.

(i) Number of clinic patients seen per month.
(ii) Time (days) between visits
(iii) 30-day/6-month all-cause/heart failure hospitalisations/mortality
(iv) Baseline to discharge changes in MARS, BMQ, KCCQ and EQ-5D-5L questionnaires. Since medication adherence and HRQoL data were not part of usual care before implementing the collaborative pharmacist medication prescribing model, data was only collected for the intervention period (March 2023–March 2024).

### Statistical analysis

All analyses were performed using Stata for Windows Version 18.0 (StataCorp. 2023. Stata Statistical Software: Release 18. College Station, TX: StataCorp LLC.) on an intention-to-treat (ITT) basis. This study was a pragmatic, real-world implementation study with our sample size reflecting all eligible patients attending the heart failure service during the study period. For this reason, we did not specify a priori sample size calculation. Continuous variables were summarised using mean and standard deviation (SD) or median and interquartile range (IQR), depending on distribution symmetry. Frequencies and percentages summarised categorical variables. Simple comparisons between the pre- and post-implementation cohorts used t-tests or rank sum tests for continuous variables, or Fisher’s exact test for categorical data. Interrupted time series (ITS) analysis modelled monthly GDMT optimisation rates (at 90 days and discharge) and clinic throughput. Optimisation at discharge, though clinically important and with potential for improvement, is a biased outcome as discharge is dependent upon optimisation. Hence, our primary outcome was optimization at 90 days as this fixed period accommodates a range of potential optimisation proportions per month and is free of such bias.

Poisson regression models with the total number of patients attending clinic per month as an offset were used to estimate level and trend changes associated with introduction of collaborative pharmacist medication prescribing. This was achieved by inclusion of a time-by-intervention interaction term, allowing estimates of immediate (main effect) and ongoing (change in slope) effects from the addition of the collaborative pharmacist medication prescribing model of care. In addition, potential seasonal effects were accounted for by the inclusion of Fourier terms, which were retained if *p* < 0.05. Autocorrelation, in general, was tested for using the Cumby-Huizinga test. [30] The effect of the collaborative pharmacist medication prescribing model was reported as the incidence rate ratio (IRR). The observation period was set at a month to provide sufficient patient numbers and allowing for at least 12 observation points before and after the intervention which is considered optimal for ITS [31, 32]. GDMT optimisation was also predicted using logistic regression with the collaborative pharmacist medication prescribing model as the predictor variable of interest, with adjustment for covariates differing between before and after the collaborative pharmacist medication prescribing periods. The effect of the collaborative pharmacist medication prescribing model was estimated as an adjusted odds ratio (OR). The effect of collaborative pharmacist medication prescribing attendance on medication adherence and HRQoL data were analysed using a generalised estimate equations (GEE) approach, assuming a normal distribution and identity link that took into consideration the correlated nature of baseline and discharge results from the same individuals. There were minimal missing data (< 10%); therefore, analyses were conducted using a complete case approach.

### Ethics approval

The study was approved by the Human Research Ethics Committees at the GCHHS and University of Queensland (Reference Number:

HREC/2023/QGC/91934) on 20^th^ April 2023. Reporting followed the Strengthening the Reporting of Observational Studies in Epidemiology (STROBE) guidelines. [33]

## Results

### Patient characteristics

A total of 302 patients were included: 98 in the pre-implementation cohort (1 December 2021 to 8 December 2022), and 204 patients post-implementation (2 March 2023 to 25 March 2024). Patient demographics at initial visit are presented in Table 2. Baseline characteristics were similar apart from a slightly higher mean heart rate in the pre-implementation group, and a higher proportion of patients taking MRAs and SGLT2-Is in the post-implementation group.

**Table 2:** Patient demographics, clinical characteristics and GDMT at baseline

Demographic variable	Pre-implementation cohort, n = 98	Post-implementation (collaborative pharmacist medication prescribing clinic), n = 204	*p* value
Age (years), mean (SD)	63 (12)	65 (12)	0.178
Male, n (%)	75 (76.5)	148 (72.5)	0.488
*Relationship status, n (%)*			
Married/partner	66 (67.4)	142 (69.6)	0.536
Single, divorced/separated, widowed	32 (32.6)	62 (30.4)	
*Heart failure aetiology, n (%)*			
Ischaemic	36 (36.7)	69 (33.8)	0.090
Arrhythmia	21 (21.4)	30 (14.7)	
Unclear cause	18 (18.4)	44 (21.6)	
Mixed cause	12 (12.3)	16 (7.8)	
Other (valvular, alcohol, drug use, chemotherapy, hypertension, viral, infiltrative, congenital)	11 (11.2)	45 (22.1)	
Ejection fraction ≤ 40%, n (%)	92, (93.9)	193, (94.6)	1.000
Mean ejection fraction, % (SD)	30.5 (8.1)	29.2 (8.6)	0.236
Mean systolic blood pressure (mmHg) (SD)	120 (20.4)	120 (18.9)	0.987
Mean heart rate (HR) (beats per minute, bpm), (SD)	75 (11.6)	71 (15.4)	0.030
Mean eGFR (mL/min) (SD)	72.9 (15.6)	70.5 (17.6)	0.253
*Comorbidities, n (%)*			
Dyslipidaemia	62 (63.3)	127 (62.3)	0.899
Ischaemic heart disease	49 (50.0)	103 (50.5)	1.000
Hypertension	41 (41.9)	93 (45.6)	0.621
Arrhythmia	47 (48.0)	74 (36.3)	0.060
Atrial fibrillation	46 (46.9)	69 (33.8)	0.032
Number of prescription medicines, mean ± SD	8 (3)	8 (3)	0.105
Guideline directed medical therapy (GDMT) at baseline, n (%)			
*Angiotensin receptor blocker + neprilysin inhibitor (ARNI)*	27 (27.8)	70 (34.3)	0.292
Angiotensin-converting enzyme inhibitor (ACE-I)/Angiotensin receptor blocker (ARB)	55 (56.1)	107 (52.5)	0.622
Beta blocker	91 (92.9)	186 (91.2)	0.824
Mineralocorticoid receptor antagonist (MRA)	34 (34.7)	115 (56.4)	0.001
Sodium glucose co-transporter 2 inhibitor (SGLT2-I)	39 (39.8)	115 (56.4)	0.010

Outcomes

Primary outcomes

Proportion of patients achieving medication optimisation of GDMT at 90-days and at discharge.

**Fig. 3:**
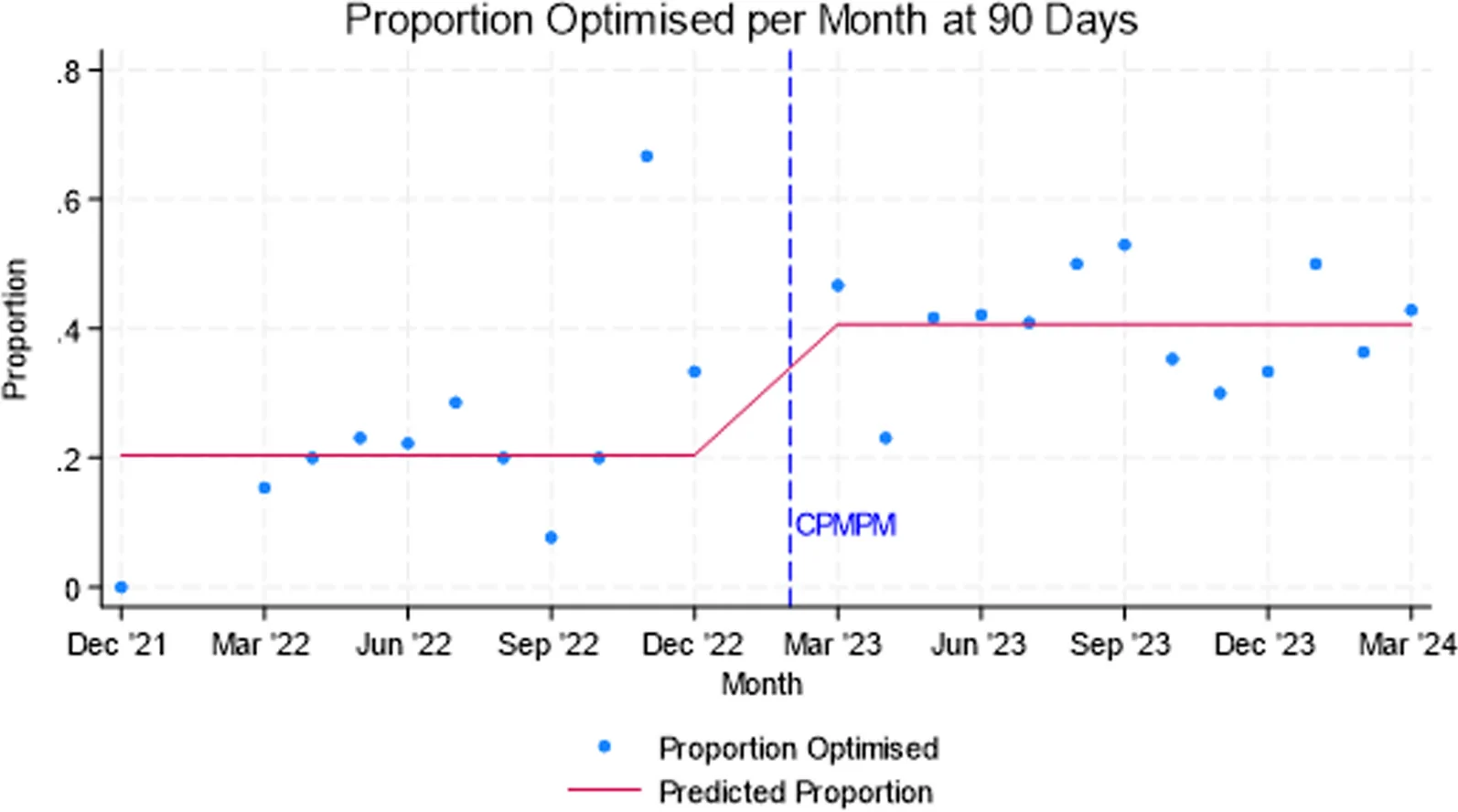
Proportion of patients optimised on GDMT 90 days before and after introducing the collaborative pharmacist medication prescribing model of care. The predicted proportion is derived from the ITS model

Interrupted time series analysis of proportion optimised at 90 days revealed no influence of time (IRR = 1.1, *p* = 0.310), time × collaborative pharmacist medication prescribing interaction (IRR = 0.92, *p* = 0.370), or seasonality (IRR 0.92 to 1.0, *p* > 0.58) presented in Fig. 3. Only collaborative pharmacist medication prescribing remained influential (IRR = 1.99, 95% CI 1.22, 3.25, *p* = 0.006). This was similarly the case for proportion optimised at discharge (time: IRR = 1.03, *p* = 0.650; time × collaborative pharmacist medication prescribing interaction: IRR = 0.97, *p* = 0.630; seasonality: IRR 0.99 to 1.1, *p* > 0.44). Collaborative pharmacist medication prescribing remained influential with IRR = 2.07 (95% CI 1.46, 2.92, *p* = 0.00004). In both cases there was no evidence of autocorrelation in the outcomes (*p* > 0.12 over 4 months). As both outcomes reduced to a simple before and after comparison of probabilities of optimisation, it was possible to perform logistic regression analyses with MRA, SGLT2i, atrial fibrillation, and baseline heart rate as adjusting covariates as they were found to differ between cohorts (Table 2). The proportion of patients on optimal GDMT increased from 19.3% (n = 19) to 40.2% (n = 82) at 90-days (adjusted OR = 2.6, *p* = 0.001), and from 43.9% (n = 43) to 88.2% (n = 180) (adjusted OR = 13.2, *p* < 0.001) at discharge. Median time to discharge from the service was similar between cohorts: 98 days (IQR 58, 154) pre-implementation and 98 days (IQR 63, 149) post-implementation (*p* = 0.980). Univariate analyses of the primary outcomes are presented in Table 3. At 90 days, target dose attainment was more frequent in the post-implementation cohort across all GDMT classes, most notably for MRAs (39.0% vs. 10.5%), presented in Table 4. Most remaining patients were receiving maximally tolerated doses below target. There was also a higher proportion of patients optimised on each of the ‘four pillars’ of GDMT at discharge in the post- compared to pre-implementation cohort.

**Table 3:** Primary and secondary outcomes (univariate analyses)

Outcome	Pre-intervention cohort, n = 98	Post-intervention cohort, n = 204	*p* value
Patients optimised on GDMT within 90 days, n (%)	19 (19.3)	82 (40.2)	< 0.001 *
Patients optimised on GDMT at discharge, n (%)	43 (43.9)	180 (88.2)	< 0.001 *
Number of new patients seen in clinic each month, mean (SD)	9 (4.23)	16 (4.35)	< 0.001 **
Number of clinic visits until optimised, median (IQR)	3 (2.0–3.5)	4 (3.0–5.0)	< 0.001 *
Days to discharge from service, median (IQR)	98 (58, 154)	98 (63, 149)	0.980***
Time between clinic visit 1 and visit 2, (days), median (IQR)	49 (35, 70)	28 (21, 42)	< 0.001 ***
Time between clinic visit 2 and visit 3, (days), median (IQR)	63 (42, 84)	28 (21, 42)	< 0.001 ***
Time between clinic visit 3 and visit 4, (days), median (IQR)	56 (35, 70)	28 (21, 44)	< 0.001 ***
Time between clinic visit 4 and visit 5, (days), median (IQR)	56 (44, 85)	28 (21, 42)	0.005 ***
Optimised on ARNI/ACE-I/ARB at discharge, n (%)	51 (52.0)	180 (88.2)	< 0.001 *
Optimised on beta-blocker at discharge, n (%)	66 (67.3)	191 (93.6)	< 0.001 *
Optimised on MRA at discharge, n (%)	67 (68.4)	178 (87.3)	< 0.001 *
Optimised on SGLT2-I at discharge, n (%)	71 (72.4)	182 (89.2)	< 0.001 *
30-day post discharge, n (%) ED visit or hospitalisation from HF ED visit or hospitalisation from any cause	1, (1.02) 6, (6.19)	1, (0.49) 13, (6.37)	0.544^*^ 1.000^*^
6-month post discharge, n (%) ED visit or hospitalisation from HF ED visit or hospitalisation from any cause	1, (1.02) 24, (24.49)	7, (3.43) 57, (27.94)	0.455^*^ 0.580^*^
6-month all-cause mortality post discharge, n (%)	0, (0.00)	6, (2.94)	0.182^*^

^*^, Fisher’s exact test; **, Welch’s t-test, ***, Wilcoxon rank sum test

**Table 4:** Optimisation of individual guideline-directed medical therapy classes at 90 days

Medication class	Pre-implementation cohort at 90 days n = 19		Post-implementation cohort at 90 days n = 82	
	Reached target dose, n (%)	Reached maximally tolerated dose*, n (%)	Reached target dose, n (%)	Reached maximally tolerated dose*, n (%)
ACE-I/ARB/ARNI	1 (5.2)	18 (94.8)	13 (15.9)	69 (84.1)
Beta-blocker	2 (10.5)	17 (89.5)	16 (19.5)	66 (80.5)
MRA	2 (10.5)	17 (89.5)	32 (39.0)	50 (71.0)

^*^maximally tolerated dose was defined as the highest tolerated dose below the guideline-recommended target dose at which further up-titration was precluded by predefined clinical criteria, documented adverse effects, or clinician assessment. SGLT2 inhibitors were not included in this table because the standard heart failure dose of dapagliflozin or empagliflozin (10 mg daily) does not require dose titration

### Secondary Outcomes

#### Number of patients seen each month

Clinic throughput increased from a mean of 9 (SD 4.23) to 16 (SD 4.35) new patients per month after introducing the collaborative pharmacist medication prescribing clinic (*p* < 0.001).

#### Time between clinic visits

Median time between visits ranged from a median of 49 days (IQR 35, 70) between early visits, to 63 days (IQR 42, 84) between later visits pre-intervention, but decreased to 28 (IQR 21, 42) post-intervention.

#### Unscheduled hospitalisations and mortality

No differences were observed in 30-day or 6-month hospitalisations or mortality (Table 3). Six patients died in the post-implementation arm and none in the pre-implementation cohort (*p* = 0.180). Four of the six patients in the post-implementation arm died from non- heart failure related causes; pancreatic cancer, septic shock associated with a diabetic foot ulcer, colorectal cancer, and glioblastoma multiforme. The other two patients were unaccounted for as they did not present to GCHHS and therefore obtaining cause of death was not possible.

#### Medication adherence, beliefs in medicines and HRQoL questionnaires

There was no change in medication adherence from baseline to discharge from the HF clinic (24.3 vs. 24.4, *p* = 0.642) in the post-implementation cohort. However, patients reported higher BMQ-necessity scores at discharge (19.5 vs. 20.6, *p* = 0.014). Improvements were also observed in patient-reported quality of life, including EQ-VAS (73.0 to 79.5, *p* = 0.004) and KCCQ scores (74.0 to 84.8, *p* < 0.01).

## Discussion

This is the first Australian study evaluating a collaborative pharmacist medication prescribing model to optimise GDMT in a community heart failure service. Our real-world findings show the collaborative pharmacist medication prescribing model of care, when incorporated into the heart failure service, increased the proportion of patients optimised on GDMT at 90 days and discharge, and shortened wait times between visits, enabling more rapid uptitration. Reducing time frames between clinic visits may have contributed to higher monthly patient throughput, freeing cardiology advanced trainees to focus on new referrals whilst the pharmacist managed ongoing titration, aligning with international evidence linking timely GDMT uptitration to reduced death or heart failure readmissions. [34]

While we saw an improvement in optimisation proportions at 90 days between the pre- and post-implementation cohorts, this was not recognized in our ITS analysis. Inspecting the monthly trends, it is likely that the large variability in optimisation proportion seen prior to introducing the collaborative pharmacist medication prescribing clinic, probably because of fewer patients seen per month, led to poor power in the ITS analysis to detect a change. After introducing the collaborative pharmacist medication prescribing clinic, optimisation rates become more consistent with a mean proportion of 40.2% at 90 days and 88.2% at discharge. This pattern suggests a possible immediate improvement in discharge optimisation with the collaborative pharmacist medication prescribing model, while the 90-day outcome appeared to follow an existing upward trend.

International registries confirm suboptimal GDMT titration globally, with only 10–30% of patients achieving target GDMT doses at 12 months and only 1% of patients receiving target doses of ‘triple therapy’ i.e. ACE-I/ARB/ARNI, beta-blocker and MRA. [35, 36] The importance of timely optimisation of GDMT was addressed in the Safety, tolerability and efficacy of up-titration of guideline-directed medical therapies for acute heart failure (STRONG-HF) trial, which demonstrated that rapid up-titration following acute heart failure admission increased achievement of GDMT targets at 90 days and reduced death or heart failure rehospitalisation compared with usual care. [34] However, important differences exist between the STRONG-HF and the present study, including patient population, study design, comparator group and outcomes. The STRONG-HF study enrolled higher-risk patients following acute hospitalisation, whereas our study evaluated a predominantly stable, community-based outpatient cohort. Furthermore, the pre-implementation comparator in our study already consisted of a specialised heart failure optimisation service rather than standard community or primary care management. As such, our findings are not directly comparable to STRONG-HF, but rather, complement existing optimisation literature by exploring the feasibility and implementation of a structured, pharmacist-led collaborative medication prescribing model of care to optimise GDMT in a real world, ambulatory heart failure service. Furthermore, our study complements or demonstrates even slightly faster rates of optimisation of GDMT compared to other community-based studies using pharmacists to optimise titration of GDMT in the UK, Canada, USA and Middle East. [8, 9, 12, 14, 37–41]

We did not observe reductions in 30-day/6-month hospitalisations, consistent with the low event rates and limited power. This differs from the STRONG-HF trial, but our pre-implementation comparator was already a GDMT optimisation service (not GP-led care, as in STRONG-HF). Stable patients (NYHA I-III) received care from the collaborative pharmacist medication prescribing clinc, whilst unstable cases remained with the advanced trainee, maintaining consistent populations and minimising bias.

We were unable to demonstrate improvements in patients’ adherence to medications, likely due to high self-reported medication adherence scores at baseline. However, increases were observed in patients’ perceived necessity of their medication and in HRQoL as reflected by changes in the BMQ, KCCQ and EQ-5D-5L scores respectively. It is important to note, however, that these validated tools were only used in the post-implementation cohort, such that we cannot directly compare to the pre-implementation cohort. Furthermore, it is not possible to determine whether these changes observed in the post-implementation cohort were attributable to the collaborative pharmacist prescribing model or reflected other aspects of HF management and recovery.

The 30-min consultations gave patients time to ask questions and allowed the pharmacist and nurse to address medication, and lifestyle concerns more effectively than shorter visits. Studies from general practice report increased patient satisfaction when given sufficient time to discuss complex health issues without feeling rushed. [42–46]. However, other literature has found no association between patient experience measures and consultation length, [47] so further research may be required to assess the impact of consultation time on quality of life measures.

One of the most influential components in establishing a successful collaborative relationship between a pharmacist and physician is trust, mutual respect and a clear understanding of respective roles. [48–50] The cardiology advanced trainees had worked alongside the pharmacist delivering the intervention over the previous 12–18 months, developing a good working relationship incorporating all of the elements listed above. Future models introducing collaborative pharmacist medication prescribing should factor this into their development to enhance chances of successful collaboration. [49, 51]

This model of care provides enhanced access to GDMT, supporting cardiologists and GPs, and could be adapted to more remote and rural settings where pharmacists could support prescribers in areas where access to health care services is more challenging. [52]

### Strengths and limitations

Strengths include real-world implementation of a mutually agreed, TIDierR protocol using a pharmacist experienced in HF management. Limitations of the study include the observational design, absence of pre-implementation medication adherence or HRQoL data, and a single-pharmacist model which limits the generalisability of the findings. Although the inclusion criterion of LVEF < 50% incorporated patients with HFmrEF, the cohort was predominantly composed of patients with LVEF ≤ 40%. Nevertheless, the findings may not be fully generalisable to populations restricted exclusively to HFrEF. Outcomes may be partly influenced by the individual pharmacist’s expertise, limiting generalisability to settings without similar pharmacist experience, training, or governance structures. Scaling up the model of care to other settings would need to address important considerations such as pharmacist skill level and competency in heart failure management, structured training pathways, workforce capacity and organisational support to ensure sustainability. Another limitation of this study was the definition of what constituted optimal GDMT. Despite using objective criteria for determining if patients were at maximal tolerated doses (e.g., heart rate, blood pressure, serum potassium, see Table 1), some degree of clinical judgement was still required when assessing suitability for further medication titration. As such, some misclassification of optimisation status cannot be excluded. Finally, we acknowledge that patients could transition between pharmacist-led and advanced trainee-led review according to clinical stability and complexity. Accordingly, the study was not intended to isolate the independent effect of the pharmacist alone, but rather to evaluate a collaborative multidisciplinary model integrating pharmacist prescribing and GDMT optimisation within an established heart failure service. The observed outcomes therefore reflect the integrated contribution of the pharmacist–doctor model of care.

### Further research

Based on the positive findings of this study, future studies should consider a more formal evaluation incorporating an assessment of the economic impact of this model of care. Whilst no apparent safety concerns were identified within the observed follow-up period, larger prospective studies are required to adequately evaluate clinical safety outcomes including hospitalisation and mortality outcomes.

## Conclusion

This single-centre, observational evaluation of a collaborative pharmacist medication prescribing model of care, appeared to be associated with higher rates of GDMT optimisation at discharge and more frequent, structured follow up for patients with stable heart failure with EF < 50%. Our findings suggest that a collaborative pharmacist medication prescribing model is feasible and may support optimisation of GDMT in a real world, Australian setting. Given the observational design, larger, controlled, multi-site studies are needed to determine whether similar models improve GDMT optimisation and clinical outcomes.

## Data Availability

The datasets generated during and/or analysed during the current study are available from the corresponding author on reasonable request.
